# CD117-Targeted Intraoperative Imaging of Gastrointestinal Stromal Tumor Using a Stem-Cell-Factor-Labeled Fluorophore

**DOI:** 10.1002/anbr.202300063

**Published:** 2023-11-27

**Authors:** Shinsuke Nomura, Shinya Yokomizo, Zhidong Wang, Homan Kang, Kai Bao, Chengeng Yang, Brian P. Rubin, Roderick Bronson, Satoshi Kashiwagi, Hak Soo Choi

**Affiliations:** Gordon Center for Medical Imaging, Department of Radiology Massachusetts General Hospital and Harvard Medical School, Boston, MA 02114, USA, Department of Surgery, National Defense Medical College, Tokorozawa, Saitama 359-8513, Japan; Gordon Center for Medical Imaging, Department of Radiology Massachusetts General Hospital and Harvard Medical School, Boston, MA 02114, USA; Gordon Center for Medical Imaging, Department of Radiology, Massachusetts General Hospital and Harvard Medical School, Boston, MA 02114, USA, Department of General Surgery, The Second Affiliated Hospital, Xi’an Jiaotong University, Xi’an 710004, China; Gordon Center for Medical Imaging, Department of Radiology Massachusetts General Hospital and Harvard Medical School, Boston, MA 02114, USA; Gordon Center for Medical Imaging, Department of Radiology Massachusetts General Hospital and Harvard Medical School, Boston, MA 02114, USA; Gordon Center for Medical Imaging, Department of Radiology Massachusetts General Hospital and Harvard Medical School, Boston, MA 02114, USA; Department of Pathology, Robert J. Tomsich Pathology and Laboratory Medicine Institute, Lerner Research Institute and Taussig Cancer Center, Cleveland Clinic, Cleveland, OH 44195, USA, Department of Cancer Biology, Robert J. Tomsich Pathology and Laboratory Medicine Institute, Lerner Research Institute and Taussig Cancer Center, Cleveland Clinic, Cleveland, OH 44195, USA; Department of Pathology, Harvard Medical School, Boston, MA 02115, USA; Gordon Center for Medical Imaging, Department of Radiology Massachusetts General Hospital and Harvard Medical School, Boston, MA 02114, USA; Gordon Center for Medical Imaging, Department of Radiology Massachusetts General Hospital and Harvard Medical School, Boston, MA 02114, USA

**Keywords:** CD117(KIT), gastrointestinal stromal tumors, near-infrared fluorophores, stem cell factors

## Abstract

Complete resection without damaging the capsule is the gold-standard surgical approach for nonmetastatic gastrointestinal stromal tumors (GIST). However, accurately locating tumors during surgery is challenging because GIST is covered by normal mucosal tissue, leading to suboptimal surgeries and increased cancer recurrence rates. To enhance surgical care for GIST, a cutting-edge near-infrared (NIR) fluorescent nanoprobe is presented that enables real-time navigation of GIST by specifically targeting CD117, a protein frequently overexpressed in GIST. By attaching a zwitterionic NIR fluorophore called ZW800–1C to a CD117 ligand, stem cell factor (SCF), precise targeting is achieved while minimizing nonspecific tissue interactions. In in vitro studies, the high affinity of nanoprobe for CD117-positive GIST-T1 cell lines is demonstrated, while exhibiting no binding to CD117-negative cells or GIST-5 R cells. In a xenograft model of GIST-T1 in mice, the nanoprobe produces strong and persistent NIR signals that last over 72 h following a single intravenous injection. Moreover, the nanoprobe successfully detects spontaneous tumors in the cecum of heterozygous Kit K641E mice. In these findings, the promise of CD117-targeted molecular imaging is highlighted as an intraoperative strategy for GIST. Furthermore, this imaging approach holds potential for early diagnosis, as well as monitoring GIST prognosis before and after surgical resection.

## 1. Introduction

Gastrointestinal stromal tumor (GIST) is the most common mesenchymal neoplasms of the gastrointestinal tract^.[1,2]^ Surgical resection is the mainstay treatment for nonmetastatic primary GIST.^[3]^ However, since GIST is a submucosal tumor derived from mesenteric stromal cells of Cajal, the tumor margin of GIST in the full thickness of mucosal tissue layers is not visible from inside or outside the gastrointestinal tract. If real-time intraoperative imaging of GIST can be realized using a specific contrast agent for GIST, surgeons would precisely be able to recognize the tumor margin and remove the tumor without damaging the capsule of the tumor during surgery, preventing the dissemination of GIST cells in the abdominal cavity. In contrast, such a specific probe would also be useful for the accurate staging of GIST. For example, metastatic GIST, especially peritoneal dissemination of GIST, is not an indication of surgery.^[3]^ In case of detection of peritoneal dissemination of GIST by intraoperative fluorescence imaging, suboptimal surgery could be avoided and chemotherapy with imatinib would be appropriately chosen to treat metastatic GIST. In short, a specific contrast agent to detect GIST is an unmet clinical need.

GISTs occur throughout the gastrointestinal tract from the esophagus to the rectum, where the smooth muscle layer or mucosal intrinsic muscle layer is located. GISTs of the esophagus, stomach, duodenum, and large intestine are often detected by endoscopy or gastrointestinal angiography, but the diagnosis of GISTs of the small and large intestine is challenging and the most promising diagnostic tool for these is multidetector CT.^[4]^ If the tumor diameter exceeds 4–5 cm on endoscopy or gastrointestinal angiography, CT or MRI should be performed to obtain a more accurate diagnosis because of the possibility of peritoneal dissemination or direct invasion of adjacent organs.^[5]^ Endoscopic ultrasound-guided fine-needle aspiration is necessary for definitive preoperative diagnosis.^[6]^ These imaging modalities for GISTs have thus far been used for preoperative diagnosis, and no solid imaging technology has been established for intraoperative guidance. However, with the spread of fluorescence-guided surgery, the molecular imaging of GISTs is becoming feasible recently.^[7]^ One of the specific molecular markers of GIST is KIT whose diagnostic sensitivity and specificity for GIST is around 85–90%.^[8]^ Therefore, KIT can be used for specific diagnoses of GIST in clinics. Stem cell factor (SCF) is an endogenous KIT ligand and can serve as a specific targeting moiety to detect GIST. Thus, bioconjugation of SCF and a contrast agent would be feasible to design a targeting construct for intraoperative imaging. However, such a conjugation strategy has not been reported to date.

Near-infrared (NIR) fluorescence imaging, in the wavelength range of 700–900 nm, has been widely used for intraoperative imaging providing sensitive, specific, and real-time trafficking of biomarkers^.[9–14]^ Unlike visible light, NIR light can penetrate sub-centimeters into living tissue due to reduced absorption and scatter; thus, exogenously administered NIR fluorescent contrast agents can be detected with high sensitivity due to low tissue attenuation and autofluorescence.^[15–17]^ Here, we report a solid strategy for molecular imaging of KIT-positive GIST using SCF conjugated with nonsticky zwitterionic NIR fluorophore, ZW800–1C,^[18–22]^ which provides minimum nonspecific tissue background, realizing specific and sensitive real-time imaging in established mouse models.

## 2. Results

### 2.1. Binding Affinity of ZW-SCF on CD117-Positive GIST Cells

**Figure 1.**
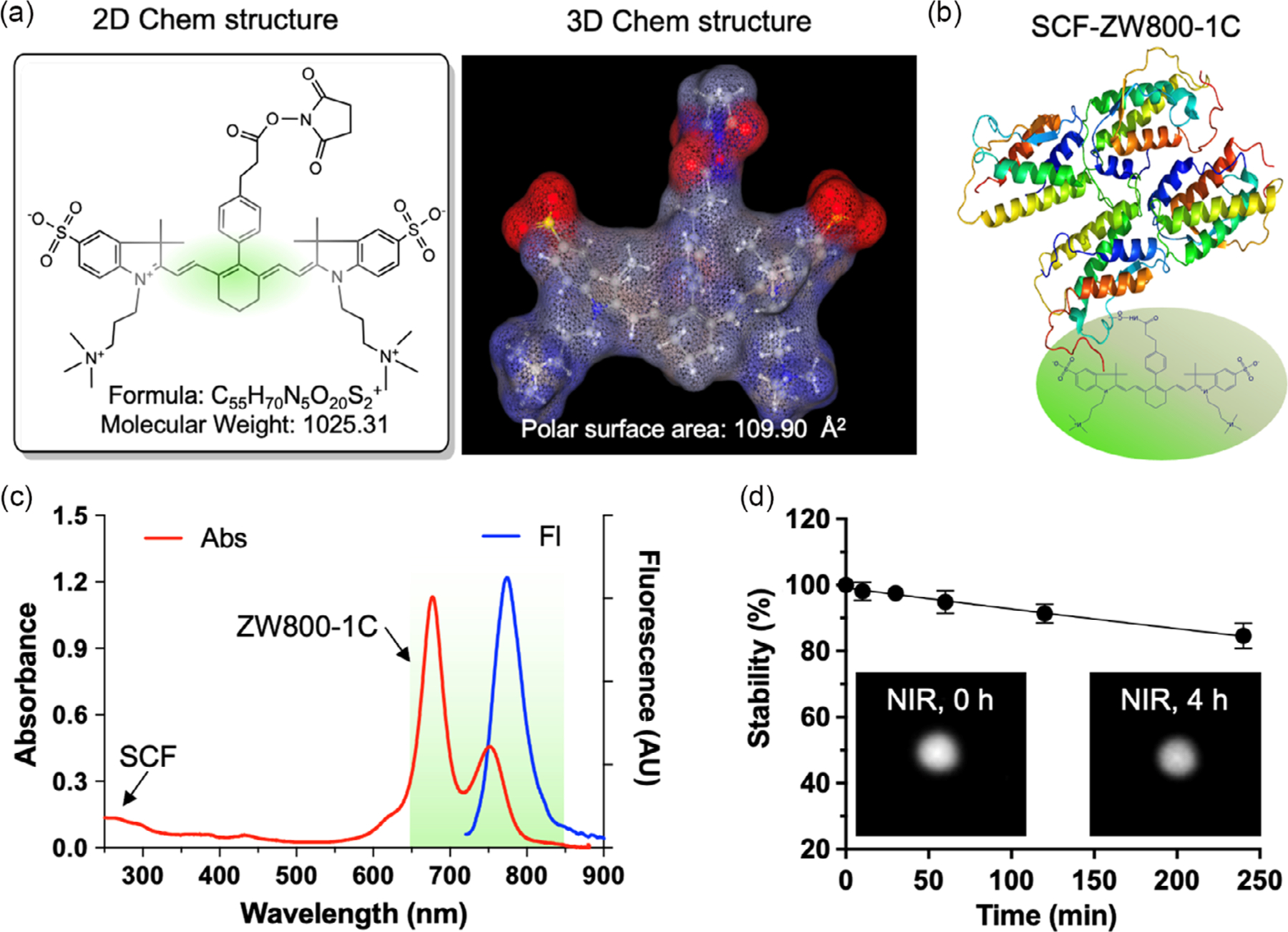
Bioconjugation of stem cell factor (SCF) with zwitterionic near-infrared (NIR) fluorophore (ZW800–1C) *N*-hydroxysuccinimide (NHS) ester. a) ZW800–1C was activated by NHS ester and b) conjugated on a lysine residue of SCF to form ZW800–1C to SCF (ZW-SCF) in phosphate buffered saline (PBS), pH 7.8. c) The optical property of ZW-SCF in 5% bovine serum albumin (BSA, 5 μM) under the UV–vis–NIR spectrophotometers. The labeling ratio of ZW800–1C against SCF was determined to be 3.42 by using Beer–Lambert’s law. d) Photostability of ZW-SCF in 5% BSA (5 μM) under the fluorescence-assisted resection and exploration imaging system for up to 4 h post-irradiation. A 760 nm excitation laser source was used at 4 mW cm^−2^ with an exposure time of 100 ms (*n* = 4, mean ± SD).

**Figure 2.**
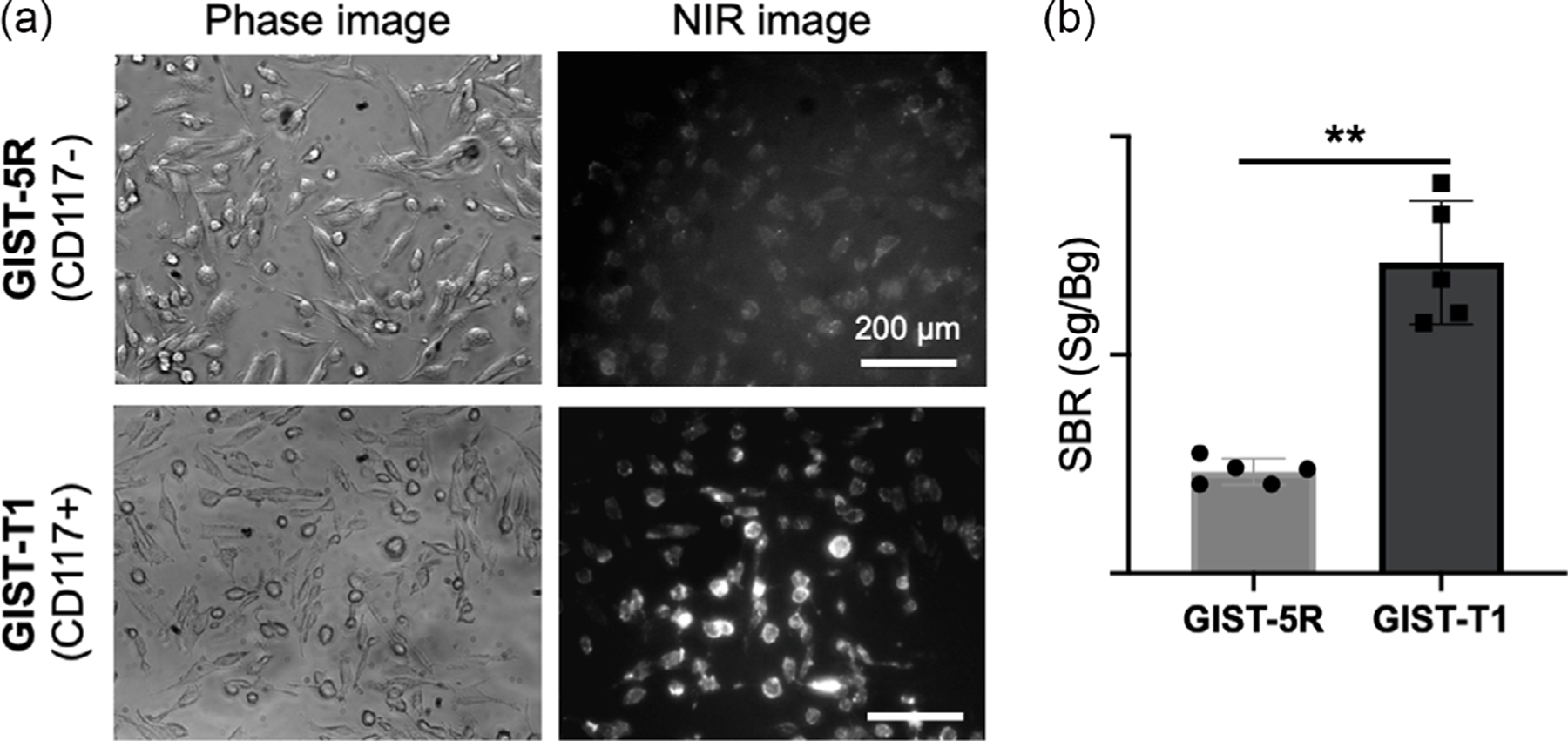
Receptor-mediated tumor uptake in gastrointestinal stromal tumor (GIST) cell lines. a) In vitro evaluation of tumor-specific uptake of ZW-SCF in GIST-5 R (CD117−) and GIST-T1 (CD117+) and b) their quantification. The signal-to-background ratio (SBR) was calculated by comparing the signal (Sg) of cancer cells against the background (Bg). **P = 0.0019, as determined by two-way ANOVA (*n* = 5, mean ± SD).

We bioconjugated the *N*-hydroxysuccinimide (NHS) ester form of ZW800–1C to SCF (ZW-SCF) to yield a specific nanoprobe for GIST (Figure 1a,b). After we purified the conjugates with a membrane filtration column, we detected robust NIR fluorescence from ZW800–1C under a custom-built NIR real-time imaging system (Figure 1c).^[23,24]^ Based on the Beer–Lambert law, we determined the labeling ratio of fluorophores on antibodies to be 3.42 calculated by absorbance measurements of SCF at 280 nm (0.12) and ZW800–1C at 760 nm (0.41) (Figure 1c). Our previous study has demonstrated that ZW800–1C shows a desirable optical property for in vivo NIR imaging and exhibits high photostability with C–C-bonded structures.^[25]^ The absorbance property of the conjugate showed little shift from the established spectrum, indicating that the bioconjugation had little effect on its optical property.^[25]^ ZW-SCF in 5% BSA solution at a concentration of 5 μM maintained about 85% (84.6% ± 3.9%) of fluorescence after 4 h of continuous NIR laser irradiation at 4mW cm^−2^ with an exposure time of 100 ms (Figure 1d). Next, we tested the binding capability of ZW-SCF to GIST cells using the NIR fluorescence microscopy in vitro. As shown in Figure 2, ZW-SCF showed binding on the surface of CD117 + GIST-T1 cells, while negligible fluorescence signals were observed in CD117–GIST-5 R cells. These results confirm the specific targeting of ZW-SCF to CD117 + GIST cells.

### 2.2. Biodistribution of ZW-SCF

**Figure 3.**
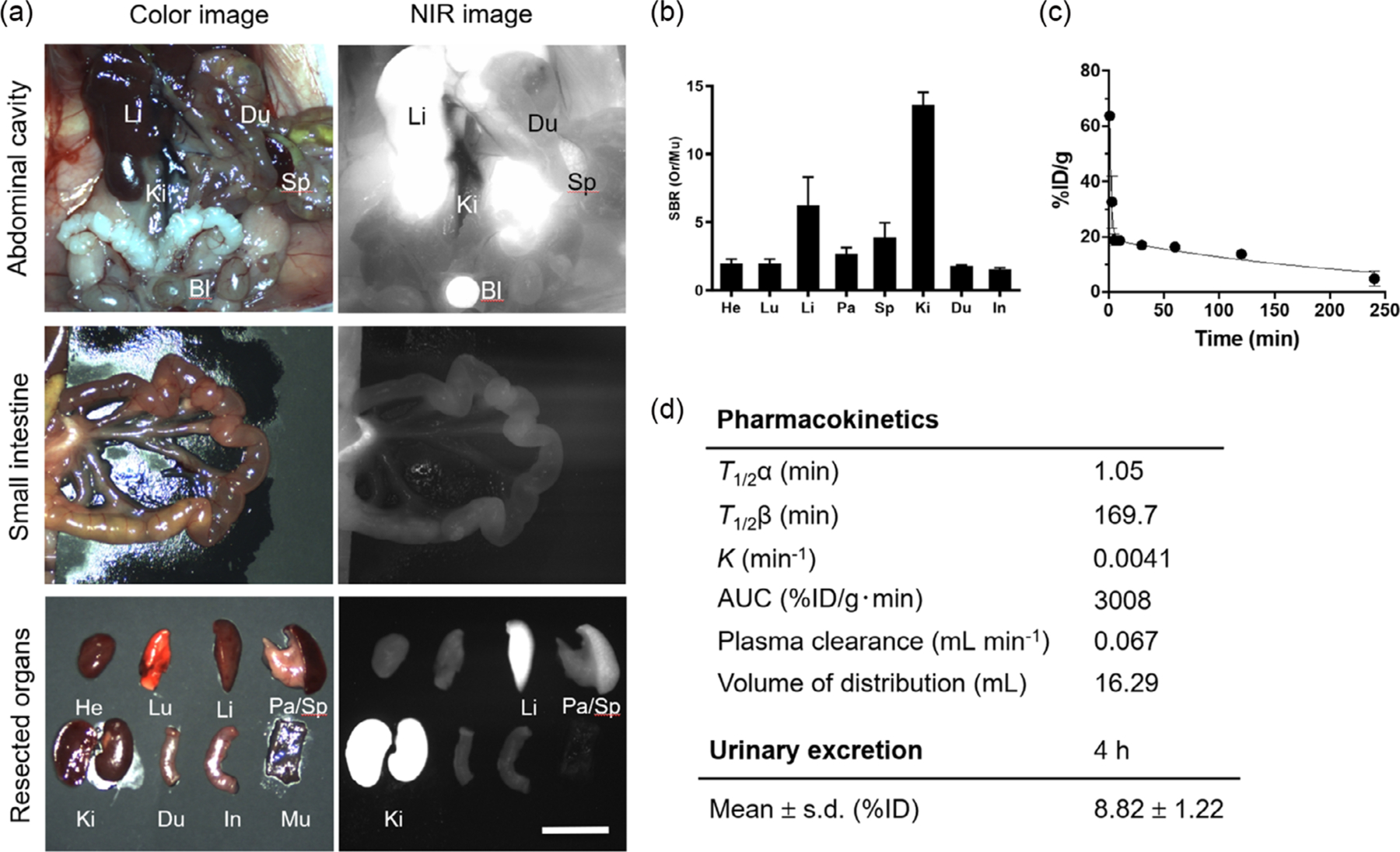
Biodistribution and pharmacokinetics of ZW-SCF. a) In vivo biodistribution and clearance of ZW-SCF. And, 1.75 nmol of ZW-SCF was injected intravenously into CD-1 mice 24 h prior to imaging. Shown are representative NIR imaging of the abdominal cavity (top), small intestine (middle), and resected organs (bottom). Scale bars = 1 cm. Abbreviations used are as follows: Bl, bladder; Du, duodenum; He, Heart; In, intestine; Ki, kidneys; Li, liver; Lu, lungs; Mu, muscle; Pa, pancreas; Sp, spleen; St, stomach. b) Biodistribution of ZW-SCF at 24 h post-injection. SBR was calculated by comparing the fluorescence signals of major organs (Or) against surrounding muscle (Mu). *n* = 3, mean ± SD. c,d) Pharmacokinetics and urinary excretion of ZW-SCF were determined in CD-1 mice injected with 1.75 nmol of ZW-SCF (*n* = 3). Blood concentration decay was obtained using capillary tubes (%ID g^−1^).

To characterize the clearance of ZW-SCF, we determined the biodistribution of ZW-SCF in CD-1 mice using the fluorescence-assisted resection and exploration (FLARE) system in vivo. As shown in Figure 3a, NIR fluorescence signals of ZW-SCF were mainly located in the liver, kidney, bladder, and spleen 24 h after injection. There were notable fluorescence signals from the kidney and liver, while little signal was observed in the intestine, suggesting a predominant role in renal clearance of the nanoprobe. The biodistribution pattern at 72 h was similar to that of 24 h (Figure S1, Supporting Information). Consistently, the signal-to-background ratio (SBR; organs vs muscle) of resected organs imaged using the FLARE system ex vivo was significantly higher in the liver and kidney (Figure 3b).

### 2.3. Pharmacokinetics of ZW-SCF

Next, we characterize the pharmacokinetics of ZW-SCF. As shown in Figure 3c, the blood concentration curve demonstrates that ZW-SCF showed a two-compartment profile of in vivo kinetics. The efficient initial distribution into capillaries reflected the rapid initial decay of blood concentration of ZW-SCF. The final concentrations reached close to the baseline at 4 h post-injection, showing rapid elimination of the conjugates from the body by systemic clearance. The half-life values of ZW-SCF were 1.05 min during the distribution phase (t1/2*α*), and 169.65 min for the terminal phase (t1/2*β*) (Figure 3d). Plasma clearance and volume of distribution of ZW-SCF after a single intravenous injection were 0.0665 mL min^−1^ and 16.29 mL, respectively (Figure 3d). Urinary excretion of ZW-SCF was determined to be 8.82 ± 1.22% ID (injected dose) 4 h after injection (Figure 3d).

### 2.4. Real-Time NIR Imaging of GIST

**Figure 4.**
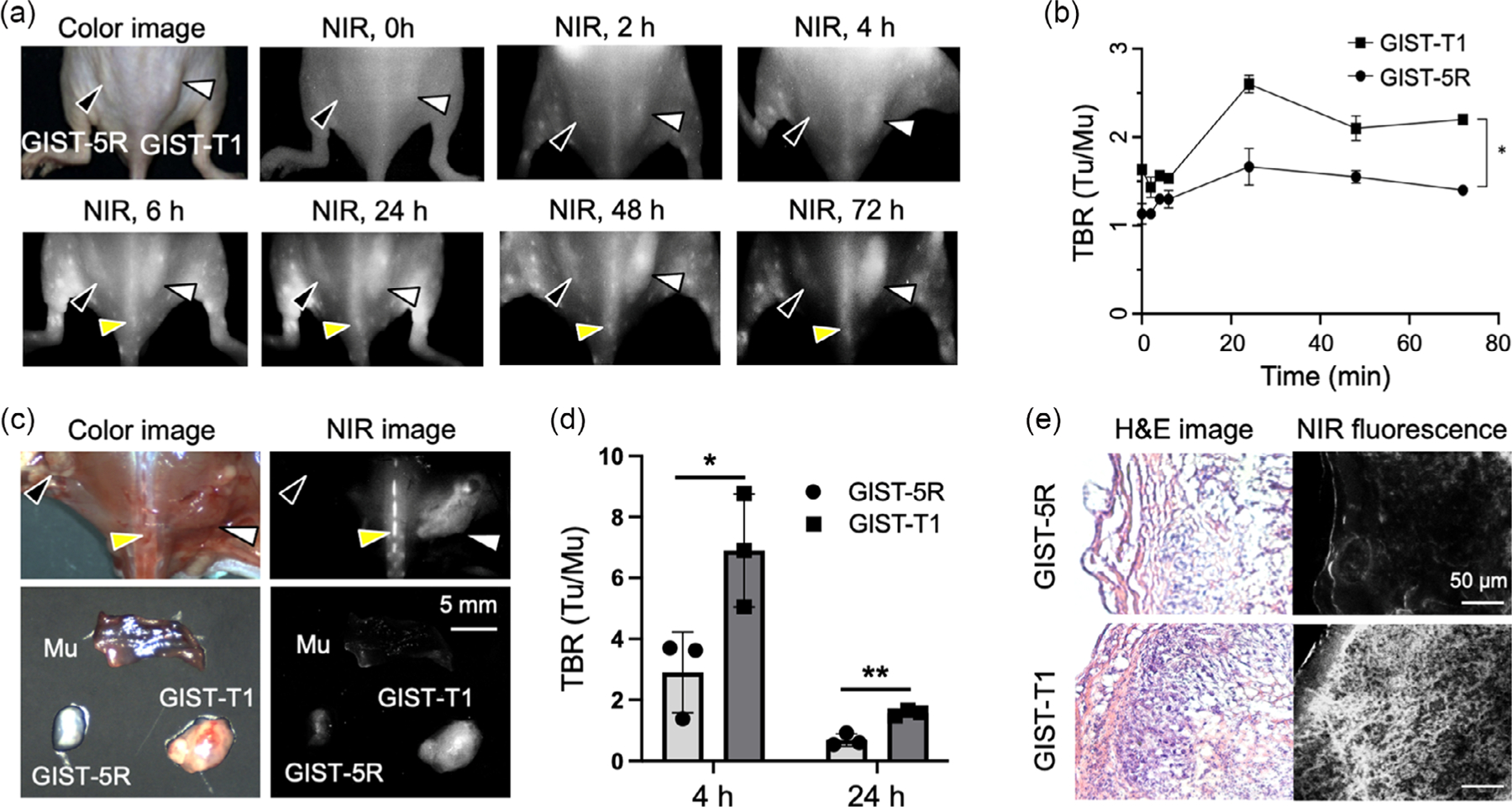
Receptor-mediated tumor uptake of ZW-SCF in xenograft tumor mice. In vivo evaluation of tumor-specific uptake of ZW-SCF in GIST-T1 and GIST-5R tumor-bearing mice. a) And, 1.75 nmol of ZW-SCF was injected intravenously into xenograft tumor mice, and NIR fluorescence imaging was performed up to 72 h. b) The tumor-to-background ratio (TBR) was obtained by comparing the signals of the tumor (Tu) against surrounding tissue (*n* = 3 per group, mean ± SD; *P = 0.0204). c) NIR fluorescence imaging of resected GIST-T1 and GIST-5R tumors. a,c) Arrowheads indicate GIST-T1 (white), GIST-5R (black), and bone marrow (yellow). d) Comparison of TBR of resected GIST-T1 and GIST-5R tumors 4 vs. 24 h. TBR was obtained by comparing the signals of the tumor (Tu) against muscle tissue (Mu) (*n* = 3 per group, mean ± SD; *P = 0.0386, **P = 0.0023). e) Histological analysis of uptake of ZW-SCF at 24 h post-injection. Slides of hematoxylin and eosin (H & E) staining and NIR fluorescence of resected tumors were imaged under the multichannel NIR fluorescence microscope. Scale bars = 50 μm.

To examine the targetability of ZW-SCF to GIST in vivo, time-course NIR fluorescence imaging of xenograft mouse models of GIST with CD117 + GIST-T1 and CD117 – GIST-5 R was performed before injection, and 2, 4, 6, 24, 48, and 72 h after intravenous injection using FLARE system. Figure 4a shows a representative time course of fluorescence images of ZW-SCF. As shown in Figure 4b, the tumor-to-background ratio (TBR; tumor vs muscle) of GIST-T1 reached the peak at 24 h after injection. While the fluorescence signal gradually decreased over time, the high TBR persisted for 72 h with the concomitant decrease in the background signal after a single intravenous injection of ZW-SCF. In contrast, the TBR of GIST-5 R also reached a peak at 24 h, but the signal was significantly lower than that of GIST-T1. Thus, we further investigated the in vivo targetability of ZW-SCF in detail at 24 h. As shown in Figure 4c, the strong fluorescence signal of ZW-SCF was detected in the resected tumor as well as the bone marrow, which is enriched with CD117+ hematopoietic stem cells. The TBR of GIST-T1 (6.91 ± 1.86) was significantly higher than that of GIST-5R (2.91 ± 1.32) (*P = 0.0386) at 4 h post-injection, which decreased to 1.57 ± 0.09 for GIST-T1 and 0.68 ± 0.21 for GIST-5R (**P = 0.0023) at 24 h, respectively (Figure 4d). Histological analysis of resected tumors using fluorescence microscopy further confirmed the specific binding of ZW-SCF to CD117 + GIST-T1 cells (Figure 4e and S2, Supporting Information). In contrast, GIST-5R tumors showed little fluorescence signal.

**Figure 5.**
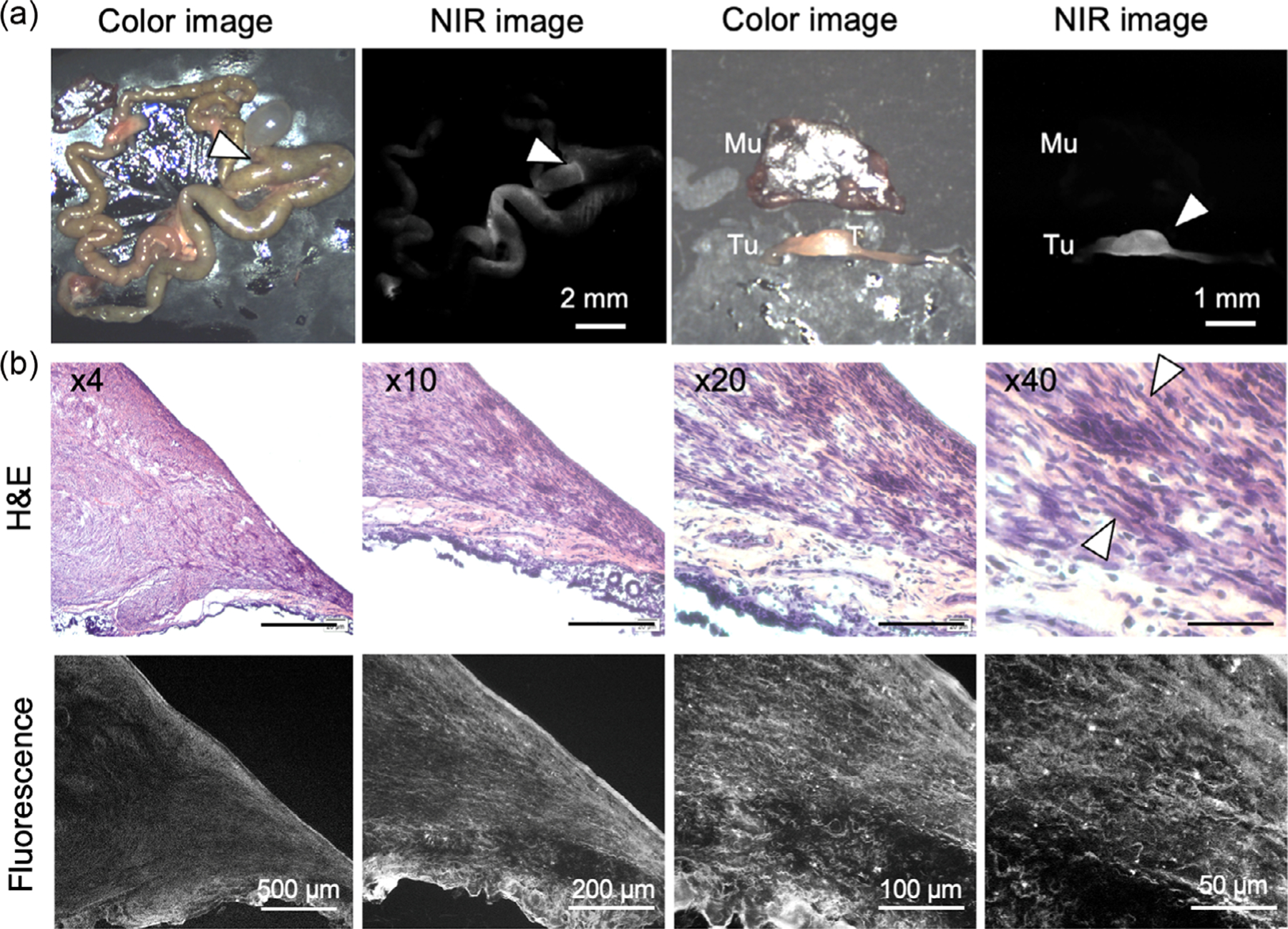
Tumor-specific uptake of ZW-SCF in spontaneous GIST model. a) And, 1.75 nmol of ZW-SCF was injected into heterozygous Kit K641E mice at 85 weeks of age, and NIR fluorescence images were obtained 72 h post-injection. The TBR was obtained by comparing the signals of tumor (Tu) against muscle (Mu) (*n* = 3–5 per group, mean ± SD; ***P = 0.003). b) Histological analysis of uptake of ZW-SCF at 72 h post-injection. Slides of H&E staining and NIR fluorescence of resected tumors were imaged under the multichannel NIR fluorescence microscope. As indicated by arrows, the GIST was identified with their fusiform spindle cells with a fascicular growth pattern.

To verify the in vivo targetability of ZW-SCF to GIST, we tested the nanoprobe using an established genetic mouse model of cecal GIST, which develops spontaneous tumors in the cecum in heterozygous Kit K641E mice. As shown in Figure 5a, the fluorescence signal from ZW-SCF was detected from the cecal GIST in this model with a high TBR (10.7 ± 2.54). As shown in Figure 5b, histological analysis confirmed high fluorescence signals co-localized with spindle-shaped GIST cells, while little signals were detected in the adjacent normal tissue. These results suggest that the nanoprobe has the potential to clarify the tumor boundary.

## 3. Discussion

In this study, we established for the first time a robust strategy for real-time fluorescence imaging of GIST using a relevant biomarker and its physiological ligand. This strategy was further validated in vivo using established mouse models of GIST. The use of nonsticky ZW800–1C provided minimum nonspecific tissue background and enabled highly sensitive real-time imaging in established mouse models of GIST up to 72 h after a single intravenous administration. Despite the demonstrated usefulness of fluorescence-guided surgery for cancer, there have been only a few reports of intraoperative fluorescence imaging of GIST to date.^[26–28]^ As a result, there is no intraoperative fluorescence imaging technology available in the clinic for GIST. The tumor models used in the previous reports have only been validated in subcutaneous tumor models or orthotopic tumor transplants. In this study, sensitive and specific imaging of GIST using ZW-SCF was further validated in a spontaneous model of GIST, which had been established to recapitulate clinical features of human GIST. Thus, our results implicate the high translatability of this imaging strategy.

Conventionally, although wedge resection for removing GIST is the standard procedure, excess normal gastric mucosa that is three times the size of the tumor has to be resected.^[29]^ Excessive resection may result in gastric deformation, or positive surgical resection margin, resulting in local recurrence.^[30]^ Hiki et al. established laparoscopy and endoscopy cooperative surgery (LECS) in 2008.^[31]^ LECS procedure is combined with the technique of endoscopic submucosal dissection to indicate the line of resected margin, and the complete resection by laparoscopic devices. LECS can remove GIST without removing excessive normal tissue, thus minimizing postoperative gastric deformation. Intraoperative NIR fluorescence imaging is compatible imaging technology with laparoscopic or endoscopic surgery. Intraoperative fluorescence imaging using ZW-SCF would assist surgeons in precisely locating GIST in a real-time manner. It would further minimize the excessive resection of the normal tissue and contribute to reducing the chance of postoperative complications.

There are three limitations to this study. First, the target receptor of ZW-SCF is KIT (CD117), which is not expressed in all GIST. Although the rate of KIT-negative GIST is ≈4%^[32]^ and quite low, it would be necessary to confirm the expression of CD117 preoperatively. Second, the optimal dose of ZW-SCF cannot be determined in this study. GIST is located under the full thickness of mucosal tissue layers and is not readily visible from inside or outside the gastrointestinal tract. The fluorescence intensity and TBR under intraoperative fluorescence imaging of GIST, which are clearly visible for operators during surgery, are not determined in the current study using small animal models. Thus, optimization of the clinical dose in large animal models that faithfully mimic the anatomy of human GIST or clinical studies is warranted. Third, activation of KIT by SCF in GIST cells stimulates several signaling pathways that promote proliferation, survival, and migration, resulting in unexpected side effects. However, small molecule imaging agents are generally too small in concentration and too transient in situ, with a half-life of 2 h only^.[33–36]^ In addition, serum concentrations of SCF that specifically activate KIT are expected to be only a transient stimulus.^[33]^ Nonetheless, the safety of this conjugate should be further determined in advanced preclinical studies and, ultimately, clinical trials.

In summary, our study successfully implemented real-time NIR fluorescence imaging for GIST by employing a targeted approach that utilizes the CD117 biomarker with SCF and nonsticky ZW800–1C. In established mouse models of GIST, the ZW-SCF conjugate exhibited favorable pharmacokinetics and enabled highly specific and sensitive intraoperative imaging of GIST for up to 72 h following a single intravenous injection, thanks to its zwitterionic coating. If validated through extensive evaluation in large animal models or clinical studies, this imaging strategy holds promising potential for early GIST diagnosis and postsurgical follow-up.

## 4. Experimental Section

### Bioconjugation of ZW-SCF:

We conjugated the NHS ester form of ZW800–1C^[25,37,38]^ with recombinant human SCF (Rockland Immunochemicals, PA), CD117(KIT) ligand protein as previously described.^[19]^ Briefly, we added 100 nmol of ZW800–1C NHS ester to 5 nmol of SCF and incubated it in 1 mL of PBS with pH 7.8 at room temperature for 3 h. We then purified the ZW800–1C-recombinant human SCF (ZW-SCF) conjugates using a mini Bio-Gel P-6 desalting column (Bio-Rad, Hercules, CA) and then concentrated the conjugates using a 10 000 molecular-weight cutoff spin column (Vivaspin 500, 10 K MWCO). We calculated the labeling ratio by determining the concentration of each compound based on the Beer–Lambert law. The extinction coefficient for ZW800–1C was 120 000 m^−1^ cm^−1^ measured at 765 nm, and SCF was 210 000 m^−1^ cm^−1^ at 280 nm, respectively. We then analyzed the optical properties of the ZW-SCF using a UV–vis–NIR spectrophotometer (USB-ISS-UV/VIS, Ocean Optics, FL).

### GIST Cell Lines:

GIST-T1 (CD117 positive) and GIST-5R (CD117 negative) cell lines were obtained from Dr. Brian Rubin at the Cleveland Clinic and grown in Dulbecco’s modified eagle medium (DMEM, Mediatech, VA) supplemented with 10% fetal bovine serum (FBS) and 1% penicillin/streptomycin in a humidified incubator at 37 °C under 5% CO2 in the air.

### GIST Cell Labeling with ZW-SCF:

To validate the specific binding of ZW-SCF to CD117 on the surface of tumor cells, we used GIST-T1 cells and GIST-T1–5R cells. We seeded cells onto sterilized glass coverslips in 24-well plates at a concentration of and incubated them for 60 min in the presence of 0.27 μM ZW-SCF at 37 °C. After gentle washing three times with 10% FBS DMEM, we imaged cells on a four-channel NIR fluorescence microscope (TE2000U, Nikon, Japan) equipped with two custom filter sets (Chroma Technologies, Brattleboro, VT) and a charge-coupled device camera (C4742–80-12AG, Hamamatsu Photonics, Japan).

### Animal:

We housed all mice in an Association for Assessment and Accreditation of Laboratory Animal Care International-certified facility at Massachusetts General Hospital. We performed all animal procedures in accordance with the Public Health Service Policy on Humane Care of Laboratory Animals and approved by the Institutional Animal Care and Use Committee (Protocol# 2016N000529). We fed all mice with a chlorophyll-free purified diet 5 days prior to imaging. We maintained mice under anesthesia by subcutaneous injection with 100 mg kg^−1^ ketamine and 10 mg kg^−1^ xylazine (Webster Veterinary, Fort Devens, MA) during the entire period of animal procedures.

### Subcutaneous GIST Model:

We purchased 6-week-old male NCr-nu/nu mice from the Taconic Farms (Germantown, NY). The GIST-T1 cells and GIST-T1–5R cells (1 × 10^7^) were suspended in 100 μL of saline/Matrigel (50 v v^−1^%) and inoculated into the subcutaneous tissue above the left and right hind leg. After the tumor size reached approximately 10 mm in diameter, fluorescent images were obtained 1, 2, 4, 6, 24, 48, and 72 h post-injection of ZW-SCF (1.75 nmol) through the tail vein. After in vivo imaging, we euthanized mice and resected major organs to histologically confirm fluorescent sites. Serial sections of OCT compound embedded tissue were prepared for hematoxylin and eosin (H & E) staining, as well as fluorescence from ZW-SCF for histological analysis.

### Spontaneous GIST Model:

Male and female Kit K641E knockin mice were kindly provided by Dr. Brian Rubin at the Cleveland Clinic.^[39]^ Heterozygous Kit K641E mice were shown to develop gastrointestinal pathology with complete penetrance. At 85 weeks, ZW-SCF (1.75 nmol) was administered 72 h prior to imaging through the tail vein, and the cecal GIST was excised and observed under the in-house built real-time intraoperative NIR imaging (FLARE) system. We performed histological analysis of the excised tumor by H&E staining, as well as fluorescence microscopy.

### Pharmacokinetics of ZW-SCF:

We purchased CD-1 mice from Charles River Laboratories (Wilmington, MA). To collect blood samples, we cut the end of the tail at a desired time point. We stored collected blood in an ice box to prevent clotting. Before injection, as a reference, we sampled blood in heparinized capillary tubes (Fisher Scientific, Pittsburgh, PA). We then injected mice with 1.75 nmol of each ZW-SCF in saline containing 5 wt v%^−1^ BSA, and we sampled blood at 1, 3, 5, 10, 30, 60, 120, 180, and 240 min to determine distribution (*t1/2*α) and elimination (*t*1/2β) blood half-life values. We centrifuged the collected blood samples for 20 min at 1000 × g to separate serum and blood plasma. We then filled capillary microtubes with supernatants of the samples. We measured the fluorescence intensities of the microtubes using the FLARE system. We presented results as a bi-exponential decay curve using Prism version 8 software (GraphPad, San Diego, CA). For the biodistribution study, we imaged mice using the FLARE system. We used a 760 nm excitation laser source (4 mW cm^−2^) with white light (400–650 nm; 40 000 lux). We acquired color and NIR fluorescence images simultaneously with customized software at rates of up to 15 Hz over a field of view (FOV) with 15 cm in diameter. After 4 h after injection, we sacrificed mice to image organs ex vivo and collected urine from the bladder.

### Quantitative Analysis of Fluorescence Images:

We quantified the fluorescence and background intensities of a region of interest (ROI) over each tissue using ImageJ v1.51 (National Institutes of Health, Bethesda, MD). We calculated the SBR as follows: SBR = *I*_ROI_/*I*_Auto_, where *I*_ROI_ denotes the average intensity of an ROI and *I*_Auto_ represents the intensity of the muscle. We calculated the TBR using the same formula, with *I*_T_ representing the intensity of the tumor tissue and *I*_B_ representing the signal intensity of the surrounding tissue: TBR = *I*_T_/*I*_B_.

### Multichannel NIR Fluorescence Microscopy of Tumor Sections:

We imaged slides on a Nikon TE2000 equipped with a QImaging color camera for bright-field imaging, Hamamatsu Orca R2 camera for fluorescence imaging, and IVision software for data collection. We obtained fluorescence images of slides with ZW-SCF using a xenon lamp passed through a 750/50 nm band-pass (BP) excitation filter, a 785 nm long-pass dichroic mirror, and an 810/40 nm BP emission filter (Chroma Technologies).

### Statistical Analysis:

All data depict the mean ± standard deviation or mean ± standard error of the mean with at least three biological replicates. We employed the student’s *t*-test to assess the statistical differences between the two groups. We used a one-way ANOVA followed by Tukey’s multiple comparisons test to compare the results among more than two groups. We considered *P* values less than 0.05 significant: *P < 0.05, **P < 0.01, ***P < 0.001, and ****P < 0.0001.

## Supplementary Material

Supplementary Material

## Data Availability

All data are available in the main text or Supporting Information.
