# Supplementary material for: CD117-Targeted Intraoperative Imaging of Gastrointestinal Stromal Tumor Using a Stem-Cell-Factor-Labeled Fluorophore
Source: Adv Nanobiomed Res. Author manuscript; Available in PMC 2025 Oct 7. (PMC12499596; doi:10.1002/anbr.202300063)
Supplement: Supplementary Material [file NIHMS2054601-supplement-Supplementary_Material.pdf]

Supplementary Information

CD117-Targeted Intraoperative Imaging of Gastrointestinal Stromal Tumor Using a Stem Cell Factor-Labeled Fluorophore

Shinsuke Nomura[1,2*], Shinya Yokomizo[1*], Zhidong Wang[1,3*], Homan Kang[1], Kai Bao[1], Chengeng Yang[1], Brian P. Rubin[4], Roderick Bronson[5], Satoshi Kashiwagi[1**], Hak Soo Choi[1**]

1. Gordon Center for Medical Imaging, Department of Radiology, Massachusetts General Hospital and Harvard Medical School, Boston, MA 02114
2. Department of Surgery and Physiology, National Defense Medical College, Tokorozawa, Saitama 359-8513, Japan
3. Department of General Surgery, the Second Affiliated Hospital, Xi'an Jiaotong University, Xi'an 710004, China
4. Departments of Pathology and Cancer Biology, Robert J. Tomsich Pathology and Laboratory Medicine Institute, and Lerner Research Institute and Taussig Cancer Center, Cleveland Clinic, Cleveland, OH 44195
5. Department of Pathology, Harvard Medical School, Boston, MA, 02115

* To whom correspondence should be addressed: HSC at hchoi12@mgh.harvard.edu or SK at skashiwagi@mgh.harvard.edu

TABLE OF CONTENTS

Figure S1. Longitudinal imaging of biodistribution of ZW-SCF

Figure S2. Figure S2. Longitudinal imaging of tumor-specific uptake of ZW-SCF in GIST-T1 and GIST-5R tumor-bearing mice.

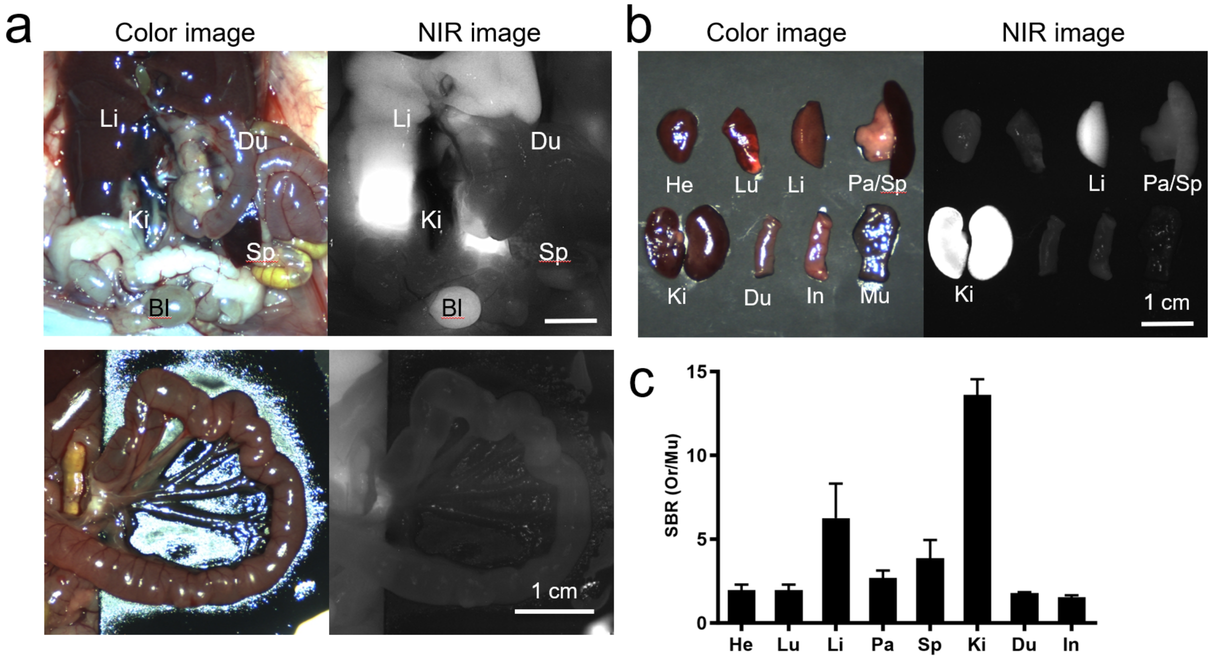

Figure S1. Longitudinal imaging of biodistribution of ZW-SCF. 1.75 nmol of ZW-SCF was injected intravenously into xenograft tumor mice, and NIR fluorescence imaging was performed up to 72 h. **(a)** Real-time imaging of abdominal cavity (top) and intestine (bottom) and **(b)** resected organs (Or) at 72 h post-injection of ZW-SCF. Abbreviations used are: Bl, bladder; Du, duodenum; He, Heart; In, intestine; Ki, kidneys; Li, liver; Lu, lungs; Mu, muscle; Pa, pancreas; Sp, spleen; St, stomach. Scale bars = 1 cm. (**c**) Signal-to-background ratio (SBR) of resected organs. SBR was calculated by comparing the fluorescence signals of major organs (Or) against surrounding muscle (Mu). n = 3, mean ± SD.

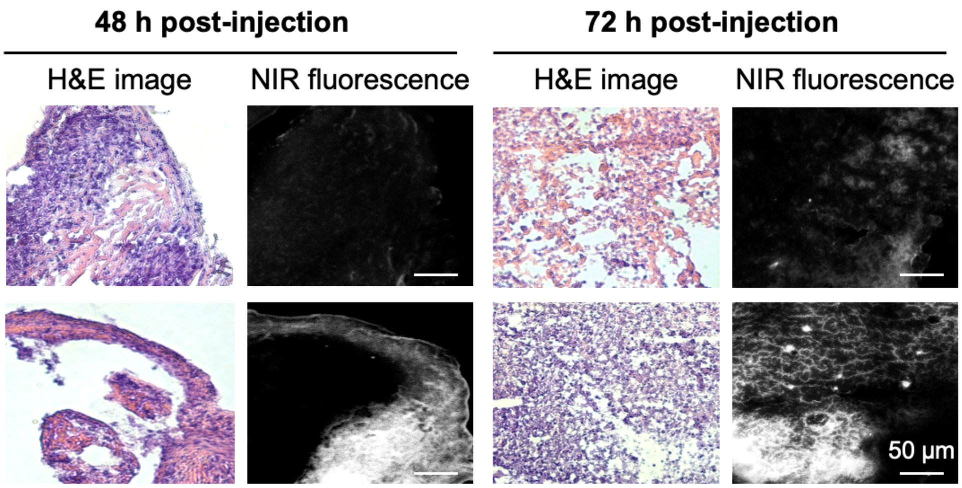

Figure S2. Longitudinal imaging of tumor-specific uptake of ZW-SCF in GIST-T1 and GIST-5R tumor-bearing mice. 1.75 nmol of ZW-SCF was injected intravenously into xenograft tumor mice, and NIR fluorescence imaging was performed up to 72 h. Histological analysis of uptake of ZW-SCF at 48 and 72 h post-injection. Slides of H&E staining and NIR fluorescence of resected tumors were imaged under the multi-channel NIR fluorescence microscope. Scale bars = 50 μm.
